# Live-streaming Activity and Relaxation Breaks: a (Home-)Office-Compatible Approach to Promote Break Recovery, Mood, and Attention?

**DOI:** 10.1007/s41542-022-00141-9

**Published:** 2023-01-12

**Authors:** Elisabeth Maria Riedl, Annabel Müller, Johanna Perzl, Joachim Thomas

**Affiliations:** 1 Department of Psychological Assessment and Intervention, Faculty of Philosophy and Education, Catholic University of Eichstätt-Ingolstadt, Ostenstraße 25, 85072 Eichstätt, Deutschland; 2 Department of Work and Organizational Psychology, Julius Maximilian University of Würzburg, Würzburg, Germany

**Keywords:** Physical activity breaks, Relaxation breaks, Recovery after break, Ambulatory attention, Within-subject field study

## Abstract

The aim of this study is to investigate whether short, live-streaming activity and relaxation lunch breaks have positive associations with office workers’ mood (calmness, valence, and energetic arousal), back pain, and attention after break and whether these associations are mediated by better break recovery. Additionally, we considered the two respite interventions as resources possibly buffering the effects of elevated situational job demands. Ten-minute break exercises were conducted during lunch breaks via Zoom live-stream, and data on those days were compared with data on days on which participants spent their breaks as usual. Our sample of 34 office workers provided data for 277 work days (209 in the home office and 68 on site at the company). Multilevel path models revealed positive total associations of both respite interventions with the mood dimension of calmness. Activity breaks additionally showed a positive association with the energetic arousal dimension of mood, while relaxation breaks were positively related to objectively measured cognitive performance. Interestingly, activity breaks moderated the relationships of job demands with calmness and valence, indicating their function as a stress-buffering resource. There were no significant associations between the two respite interventions and back pain. Supplemented by participants’ feedback, the findings of this study suggest that offering short virtually guided break exercises may represent a feasible and office-compatible approach to promote break recovery, mood and functionality at work, especially regarding home-office work. Possible advantages and disadvantages of the live-streaming format are discussed.

## Introduction

Contrary to the belief that office work represents a low-stress job (BAuA, 2010), many office workers are frequently exposed to high job demands, especially multi-tasking (68%), time and performance pressure (48%), and frequent interruptions (48%). Compared to numbers reported in surveys in 2006 and 2012, the number of office workers feeling overwhelmed by quantitative job demands has increased (BAuA, 2020). “Quantitative job demands constitute those elements of the work environment that concern the amount and speed of work to be performed, and require physical and/or psychological effort” (van Veldhoven, 2014, p. 121). During the COVID-19 crisis, many office jobs shifted to the home office, which added further challenges related to, for example, poor ergonomic conditions at home (Bouziri et al., 2020).

The experience of chronic stress due to high job demands is associated with negative health consequences in the long term (Meijman & Mulder, 1998). Nearly 80% of office workers report at least one musculoskeletal or psychovegetative complaint (BAuA, 2020). To prevent negative long-term consequences from job stress, work breaks are increasingly moving into the focus of organizational research (Scholz et al., 2019). Work breaks serve to restore exhausted resources (Wendsche & Lohmann-Haislah, 2016). Generally, work breaks prevent fatigue, help maintain attention, and promote the recovery of health and performance (Wendsche & Lohmann-Haislah, 2016). However, there are many different types of work breaks. The types of work breaks most suitable for the recovery of depleted resources depend on the job demand profile and its psychological and physiological consequences.

### Purpose of the Present Study

To promote recovery from high mental demands and sedentary work in the office, relaxation exercises and physical activity are recommended (Wendsche & Lohmann-Haislah, 2016). The central purpose of this study is to investigate whether short, live-streaming activity and relaxation lunch breaks have positive associations with office workers’ mood (calmness, valence, and energetic arousal), back pain, and attention after break and whether these associations are mediated by better break recovery. To our knowledge, no intervention study has investigated live-streaming break activities among employees. However, as the pandemic-related home office boom is expected to have a lasting impact on the organization of work toward an increase in teleworking (Alipour et al., 2020), break offers that do not require physical presence at the office are increasingly important.

Lasting eight to ten minutes, we deliberately kept the break interventions short. Steidle et al. (2017) defined a respite intervention “as a micro-intervention with a length of 5–10 min that can be completed at the workplace and gives an employee a reprieve from work, during which employees shift their attention away from work tasks” (pp. 650–651). Several studies have investigated break interventions at the workplace, but research on short break interventions is particularly scarce. Applying the definition of respite interventions from a daily perspective, with the exception of the intervention study by Steidle et al. (2017), none of the intervention studies that we found used a respite intervention in the strict sense, as the daily intervention duration exceeded 10 min. If short respite interventions were nevertheless effective, they would bring important advantages over other break interventions. The low intensity and high office compatibility of a respite intervention (Steidle et al., 2017) is likely to appeal to more people in the target group and increase the applicability of the intervention in different occupational contexts. Furthermore, in the long term, a respite intervention is probably more successfully implementable than longer interventions, as the “light” format (Steidle et al., 2017, p. 660) leaves time for other activities that people normally engage in during their breaks. In addition, and thematically important for this study, a respite intervention of up to 10 min should be feasible even on busy days, when employees tend to skip or shorten their breaks (BAuA, 2015).

Sonnentag et al. (2017) drew attention to a paradox concerning the associations between job demands and recovery activities and experiences. They concluded from their review that high job demands hinder recovery activities and experiences, but under high-stress conditions, it is particularly important to replenish depleted resources by engaging in recovery-promoting activities. We assume that the offer of virtually guided break activities may be particularly helpful on days with elevated job demands because on high-stress days, employees’ usual break activities are suboptimal (Sonnentag et al., 2017). However, as potentially stress-buffering resources must be useable in high-stress situations, we examine short respite interventions. Investigating whether virtually guided break activities can be a resource with the potential to function as a demand buffer, we address the need to investigate moderating variables in the context of break interventions (Steidle et al., 2017).

This study uses a within-subject field design to address the question of whether the same group of office workers show better break recovery, mood, and attention and lower back pain on days on which they participate in activity and relaxation breaks than on days where they spend their breaks as usual. The most important advantages of this research method are its high ecological validity and its effective control of individual differences.

Furthermore, most studies that investigate break interventions have focused on the selected subjective experiences of participants. As work breaks influence affective, cognitive, and physiological processes, work break research should consider multiple outcomes and ideally combine self-report data with performance or physiological measures (Scholz et al., 2019). Correspondingly, we assessed self-reported psychological and physiological data and combined it with objectively measured attention to address the need to include objective data (Kim et al., 2017; Sonnentag et al., 2017).

### Breaks from the Perspective of the Effort-Recovery Model and Conservation of Resources Theory

According to the effort-recovery model (ERM; Meijman & Mulder, 1998), employees meet work demands by engaging in work activities that trigger short-term physiological and psychological reactions. These adaptive reactions can be reversed by recovery, the process in which psychobiological systems return to baseline levels. For successful recovery, it is critical that stressed psychobiological systems have a chance to rest, which requires that work demands no be longer present. When work demands persist and recovery is insufficient, negative load effects may occur, which include a longer-term and potentially irreversible impairment of health and well-being due to permanent pathological changes in the psychobiological systems involved (Meijman & Mulder, 1998).

Similarly, in conservation of resources (COR) theory (Hobfoll, 2002), coping with work demands consumes resources such as energy and cognitive capacity. In a state of depleted resources, people are motivated to restore their resource pool. A prompt and successful recovery of resources is important because resource loss is associated with stress experiences, which in turn depend on the availability of resources. Thus, the vulnerability of individuals increases when they are confronted with resource-consuming demands before their resource pool is replenished. To prevent resource loss cycles (Hobfoll, 2002), it may be beneficial to engage in activities that allow a prompt revitalization of the resource pool. Based on the assumption that simply taking a break may not be sufficient, Trougakos and Hideg (2009) differentiated two types of break activities that differ in their demands and control. Characterized by low effort and preferred choice/enjoyability, respite activities stop the process of resource depletion and provide useful resources that support well-being and work capability (Trougakos & Hideg, 2009; Hunter & Wu, 2016) showed that preferred activities during breaks are positively associated with self-reported post-break energetic, attentional, and motivational resources. Chong et al. (2020) suggested that workday respite activities are affective events that, following affective events theory (Weiss & Cropanzano, 1996), trigger positive affective states, which promote motivation, mitigate negative affective states and limit strain. While relaxation represents a typical example of an enjoyable low-effort activity, physical exercise can also be a respite as long as it is an individual choice. Chores, on the other hand, are activities on a to-do list. Whether work-related or not, they are experienced as effortful and are usually not a preferred activity; thus, they draw on self-regulatory resources and interfere with the pursuit of preferable break activities (Trougakos & Hideg, 2009).

From the ERM perspective (Meijman & Mulder, 1998), it is crucial that work breaks be free from work demands. Work-related lunch break activities are associated with higher negative affect after a break and a lower perceived person-break fit, which relates to the degree to which breaks match individual needs and preferences (Venz et al., 2019). Offering guided break activities promotes the passive aspect of breaks by ensuring that at least part of the break is not used for continuing work-related tasks. Relating to the active aspect of breaks, resource-building and revitalizing activities represent particularly suitable content (Hobfoll, 2002).

### Empirical Evidence Regarding Activity and Relaxation Breaks

Empirical evidence indicates that physical activity and relaxation may represent resource-revitalizing activities that actively promote recovery from demands. Cross-sectionally, light (e.g., stair climbing) and moderate (e.g., walking during lunch breaks) physical activities at work are associated with a lower need for recovery (Coffeng et al., 2015). Taking an experimental approach, Hoover et al. (2022) recently showed that a physical activity break after induced resource depletion resulted in higher energy levels than relaxation breaks, while relaxation breaks resulted in higher detachment and relaxation than the physical activity break. Overall, energy levels after recovery break activities lasting 15 min returned to baseline, indicating the replenishment of lost resources. Additionally, after the break, self-regulatory capacity increased and fatigue declined beyond baseline levels, suggesting that recovery break activities may even facilitate resource accumulation (Hoover et al., 2022).

In an early intervention study by Kennedy and Ball (2007), audio-instructed hypnorelaxation power tea breaks were related to a reduction in fatigue, negative mood, and physical symptoms and an increase in job satisfaction compared to a control group that spent the two tea breaks each day as usual. Krajewski et al. (2010) showed that a 20-minute, audio-instructed progressive muscle relaxation lunch break was associated with a reduction in strain states among call center agents compared to a control group that took short talk breaks. A recent randomized-controlled study (Díaz-Silveira et al., 2020) compared 30 min of audio-guided mindfulness mediation and supervised physical exercises during lunch breaks with a control group. After four weeks of training, the physical exercise group showed lower perceived stress scores than the control group. The mindfulness mediation group benefitted in the overload subdimension more than the control group. Sianoja et al. (2018) investigated the within-person relationships of 15-minute park walks and relaxation exercises within lunch breaks. Both groups showed improvements in afternoon strain on intervention days than on non-intervention days, and the relaxation group additionally showed a decrease in afternoon fatigue on intervention days. Interestingly, both intervention groups reported better concentration on intervention days.

Steidle et al. (2017) conducted a 4-week longitudinal field experiment that used respite interventions within lunch breaks among administrative and knowledge workers. The intervention groups engaged in a short, simulated nature-savoring intervention or progressive muscle relaxation. Indicating an upward resource trajectory, in the respite intervention group, vigor increased and fatigue declined over the course of the intervention. Interestingly, daily resource gains in the intervention group translated into more stable resource gains in terms of higher post-intervention vigor and lower exhaustion.

In this study, we applied a within-subject field design to investigate the associations of activity and relaxation breaks with break recovery, mood, attention, and back pain compared to breaks spent as usual. As work breaks influence affective, cognitive, and physiological processes, work break research should consider multiple outcomes and ideally combine self-report data with performance or physiological measures (Scholz et al., 2019).

Based on the three-dimensional affect model (Matthews et al., 1990; Schimmack & Grob, 2000), we assessed the associations of the activity and relaxation breaks with self-reported daily mood in terms of valence, calmness, and energetic arousal. Valence refers to the affective tone (from unpleasant to pleasant), calmness refers to the tension level (from agitated to calm) and energetic arousal refers to wakefulness and energy (from tired to awake; Schimmack, 1999). Wilhelm and Schoebi (2007) showed that these three theoretically postulated, correlated dimensions are necessary to describe within-person variations of mood and exhibit large proportions of within-subject variances that indicate high sensitivity to change. The content of the relaxation breaks is directly linked to the calmness dimension, whereas the activity breaks were designed to activate the participants and promote energy and suggest a strong association with energetic arousal. Both break types should be perceived as enjoyable break activities and thus be associated with the valence dimension.

Beyond benefitting mood, a successful restoration of resources should also benefit the attention performance after break, because during work, cognitive capacity resources are invested (Hobfoll, 2002). Indeed, intervention studies have found positive associations between relaxation exercises and physical activity with self-reported concentration (Krajewski et al., 2010; Sianoja et al., 2018). Finally, break interventions may also be helpful to lower physical strain states (Krajewski et al., 2010) such as back pain. Based on the theoretical arguments and prior empirical findings, we propose the following hypotheses:

- H1: On days on which office workers engage in live-streaming activity breaks, calmness (H1a), valence (H1b), energetic arousal (H1c) and attention (H1d) are higher and back pain is lower (H1e) after breaks than on days on which breaks are spent as usual.
- H2: On days on which office workers engage in live-streaming relaxation breaks, calmness (H2a), valence (H2b), energetic arousal (H2c) and attention (H2d) are higher and back pain is lower (H2e) after breaks than on days on which breaks are spent as usual.

Due to their promotion of passive and active recovery, we assumed that both break intervention types are generally effective in replenishing affective, energetic, attentional, and physical resources, and we did not specify different associations for activity and relaxation breaks. Thus, we suggest that the break recovery experience plays a central mediating role in the associations of activity and relaxation breaks on the one hand and mood, attention, and back pain on the other hand. Recovery experiences are supposed to be crucial for health, well-being and performance capability (Sonnentag & Geurts, 2009). Compared to recovery after work, recovery during work has received much less attention in the research (Díaz-Silveira et al., 2020).

In both contexts, recovery experiences are regarded as a mediator between work characteristics and well-being (Bennett et al., 2018; Chong et al., 2020; Sianoja et al., 2018). The mediating role of workday recovery experiences was investigated by Chong et al. (2020), who found that the recovery experience detachment mediated the associations of workday respite activities on positive and negative affectivity. In an intervention study by Sianoja et al. (2018), the recovery experience enjoyment mediated the associations of park walks with fatigue and subjective concentration, while for relaxation, indirect associations with concentration via detachment were found.

**Fig. 1:**
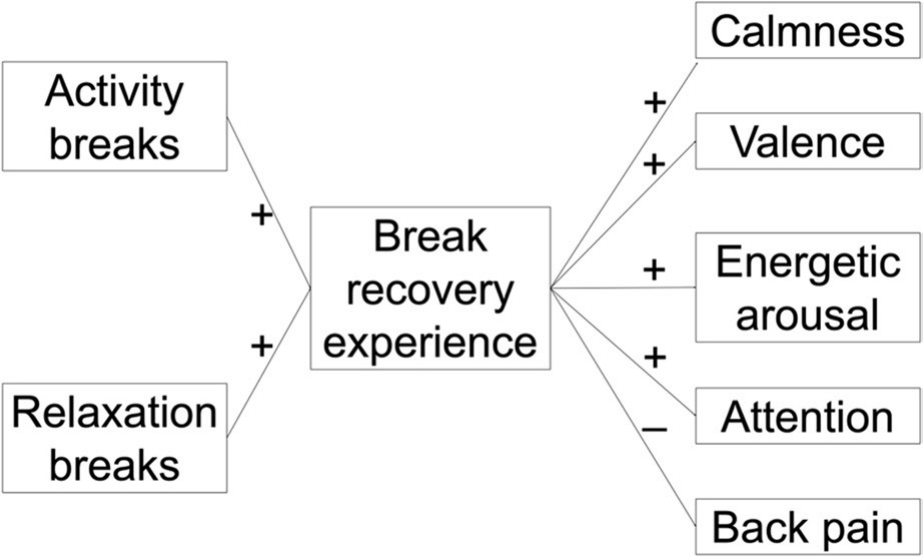
Graphical illustration of the proposed mediation model

Recovery experiences are often studied (Bennett et al., 2018; Headrick et al., 2022; Steed et al., 2021) in the framework of Sonnentag and Fritz (2007), who proposed the four recovery experience dimensions of detachment, relaxation, mastery, and control. Demerouti et al. (2012), however, proposed a general construct of the recovery experience at work, which refers to the degree to which individuals perceive that a break was helpful for recovery and the restoration of energy resources. The general recovery experience at work was positively correlated with vigor at work and negatively correlated with exhaustion (Demerouti et al., 2012). Relating to the mediating role of break recovery experiences, we propose the following hypotheses, which are graphically illustrated in Fig. 1.

- H3: Mediated by the break recovery experience, there are significant positive indirect associations of activity breaks with calmness (H3a), valence (H3b), energetic arousal (H3c), and attention (H3d), and there is a significant negative indirect association of activity breaks with back pain (H3e).
- H4: Mediated by the break recovery experience, there are significant positive indirect associations of relaxation breaks with calmness (H4a), valence (H4b), energetic arousal (H4c), and attention (H4d), and there is a significant negative indirect association of activity breaks with back pain (H4e).

### Respite as a Buffer Against High Job Demands

The job-demands resources theory (JDR; Bakker & Demerouti, 2017) differentiates two categories of characteristics of the working environment: job demands and job resources. Job demands are experienced as effortful and associated with physiological and/or psychological costs, which can lead to stress, depletion and health complaints. Resources, on the other hand, represent factors that support goal attainment, reduce job demands or their physiological and psychological costs, or stimulate personal learning and development. The buffer hypothesis of the JDR states that job resources are able to mitigate the positive relationship between job demands and indicators of strain. The buffer effect is theoretically derived from the COR (Hobfoll, 2002). Coping with demands costs resources, but when this resource loss is compensated by other resources, the resource pool and thus well-being remain balanced. The definition of job resources suggests that breaks in general, and respite interventions in particular, can be understood as resources, as they aim to reduce demands during break time. Furthermore, respites are helpful in recovering and increasing energy levels (Steidle et al., 2017) and reducing strain reactions (Sianoja et al., 2018). Indeed, one study supported the assumption that breaks may function as a demand buffer. Taking a within-subject perspective, Kim et al. (2017) showed that relaxation and socialization microbreaks during the afternoon buffered the positive relationship between elevated quantitative job demands and negative affect at the end of the work day, while relaxation also included light forms of physical activity such as stretching or walking.

However, high job stressors predict low detachment, and detachment is crucial for the recovery process (Sonnentag & Fritz, 2015). Sonnentag et al. (2014) showed that time pressure intensifies the negative relationship between exhaustion and detachment. Therefore, it can be assumed that without intervention, people have difficulties recovering during work breaks when they have to deal with elevated situational job demands, which is when effective rest is particularly important (Sonnentag et al., 2017). However, a respite intervention may be able to counter this paradox. Therefore, we propose the following hypothesis:

- H5: On days on which office workers engage in live-streaming activity breaks, the association between quantitative job demands and calmness (H5a), valence (H5b), energetic arousal (H5c) and attention (H5d) is less negative, and the association between quantitative job demands and back pain (H5e) is less positive after breaks than on days on which breaks are spent as usual.
- H6: On days on which office workers engage in live-streaming relaxation breaks, the association between quantitative job demands and calmness (H6a), valence (H6b), energetic arousal (H6c) and attention (H6d) is less negative, and the association between quantitative job demands and back pain (H6e) is less positive after breaks than on days on which breaks are spent as usual.

## Method

### Sample and Procedure

This study was conducted in June and July 2021 among office workers of a large industrial company in Germany. The employees were invited to participate in the study via email. Interested employees registered by email and were sent the participation materials via postal mail to their home addresses. The study was conducted on 15 working days from Monday to Thursday with alternating activity, relaxation and control breaks. We did not collect data on Fridays because many employees finish early on Friday. Activity breaks, relaxation breaks and control breaks were alternated so that the same condition did not come twice in a row. The reasons were to control the exercise effects on the one hand and to provide variety on the other hand. Furthermore, we balanced the conditions regarding the working days, because some participants may be confronted with recurring demands on some working days, e.g., a demanding meeting on Wednesday. The study started with activity breaks, then there was a control day followed by a relaxation break. To provide the trainers and participants a certain orientation, the same order of the three conditions was maintained.

In the morning, the participants received a reminder mail with instructions for the day. On control days, the participants were instructed to spend their breaks as usual. On intervention days, sport or relaxation therapists on the company’s health management team guided participants through the activity or relaxation break via a Zoom live stream at 1 pm. The sessions lasted between eight and ten minutes. Oriented towards Yin Yoga (Arend, 2017), the relaxation breaks included mindful breathing exercises, stretching, self-massages and eye relaxation exercises. The activity breaks focused on strengthening the trunk and back muscles and improving mobilization and coordination. On each study day at 1:15 pm, a smartphone alarm reminded participants to complete the daily survey, which lasted approximately four minutes and could be completed before the end of the work day. The mean time at which participants completed the daily questionnaire was 1:55 pm (*SD* = 1:53). At the beginning of the study, the participants answered a short one-time smartphone survey that collected demographic information and provided a demonstration of the attention test. The study ended with a short feedback survey.

Thirty-nine employees registered for the study. Five employees were excluded because they did not fulfill the criteria of participating and providing valid data on at least two study days. The remaining 34 employees provided 277 valid daily measurements (97 relaxation days, 103 activity days, and 77 control days), which corresponds to a compliance rate of 54%. On 209 days, the participants worked from home, and on 68 days, they worked in the office. The demographic information for the sample is presented in Table 1.

**Table 1:** Demographic information for the sample

Category	Frequency
Sex	
Male	10
Female	24
Diverse	0
Age	
< 35 years	15
35–44 years	8
> 44 years	11
Standard working time	
Fulltime	29
Part-time (> 20 h per week)	5
Part-time (< 20 h per week)	0

### Measures

Unless stated otherwise, all variables were measured on a seven-point Likert scale from *strongly disagree* (1) to *strongly agree* (7).

#### Job Demands

The Stress Report (BAuA, 2020) indicates that (1) facing time and performance pressure and (2) working on different tasks at the same time constitute the main psychological job demands for office workers. Both aspects relate to quantitative job demands, which are the most often investigated job demands across occupations (Peeters et al., 2005). Referring to the German version (Nübling et al., 2005) of the Copenhagen Psychosocial Questionnaire (COPSOQ; Kristensen et al., 2002), facing time pressure was assessed with the item “Today, I was under time pressure”, and working on different tasks was assessed with the item “Today, I had to be attentive to many things at the same time” (see Riedl & Thomas 2019). Both items showed a within-subject reliability (Nezlek, 2011) of 0.73.

#### Recovery Experience

The break recovery experience was assessed with the three-item short scale of Demerouti et al. (2012). The items were introduced by “Please answer the following statements about your break”. A sample item is “During my break I could recuperate” (cf. Demerouti et al., 2012, p. 282). The scale showed a within-subject reliability of 0.83.

#### Calmness

Calmness was assessed with the two-item short scale of Wilhelm and Schoebi (2007), which is based on the Multidimensional Mood Questionnaire (Steyer et al., 1997) and uses two seven-point bipolar items for each of the three mood dimensions. For calmness, the two items are “agitated-calm”, and “relaxed-tense” (Wilhelm & Schoebi, 2007). They were introduced by the question “At the moment, I feel…”. At the within-subject level, both items showed a reliability of 0.53.

#### Valence

Relating to the present moment, valence was measured by the two bipolar items “content-discontent” and “unwell-well” from the daily version (Wilhelm & Schoebi, 2007) of the Multidimensional Mood Questionnaire (Steyer et al., 1997). The within-subject internal consistency of the valence scale was 0.43.

#### Energetic Arousal

Momentary energetic arousal was measured by the two bipolar items “tired-awake” and “full of energy-without energy” from the daily version (Wilhelm & Schoebi, 2007) of the Multidimensional Mood Questionnaire (Steyer et al., 1997). The within-subject reliability coefficient was 0.67.

#### Back Pain

We assessed the following six somatic symptoms by items adapted from the Symptom Checklist Revised (Derogatis, 1977): headache, dizziness, back pain, difficulty catching breath, numbness/tingling, and muscle soreness. The items were introduced by the question “To what extent are you currently experiencing…”. However, at the situational level, the items showed only small to moderate intercorrelations (*r* = .11 to *r* = .53). Therefore, we report the results only for back pain, which achieved the highest mean (*M* = 2.19). Generally, the main results for the other somatic symptoms were similar to the results for back pain and can be obtained from the authors upon request.

#### Attention Measurement

Attention was assessed by the sustained attention to a response task (Robertson et al., 1997), which was a go/no-go task constructed to measure everyday attention failures. In this test, the task is to react to every digit from 1 to 9 except 3 by touching a screen (digit presentation time: 250 ms, mask: 900 ms). The original version of the test includes 25 passes of digits 1 to 9, which corresponds to a test duration of 4.3 min. To avoid overburdening the participants during the repeated measurement, the test was reduced to 12 passes. However, due to technical problems, eleven participants received the original version instead of the shortened version. Therefore, we used the percentage of errors committed as the outcome variable. This outcome variable was not influenced by the test length (estimate = 0.41, *CRI* 95% [-1.26, 1.17]), and the results did not change when the test length was controlled.

### Data Analysis

The data showed a hierarchical structure, with measurements nested within persons. Therefore, multilevel models were built to take the dependency in the data set into account. The statistical analyses were performed with MPlus version 8.1.6. Indicating the need for multilevel models, all dependent variables showed significant within-subject and between-subject variances. The intraclass correlations ranged from 0.25 (calmness) to 0.67 (back pain; see Table 2). Compared to the break recovery experiences and the three dimensions of daily mood, which showed very large day-level variance, for back pain and the percentage of commission errors, the between-subject differences outweighed the day-level differences.

**Table 2:**
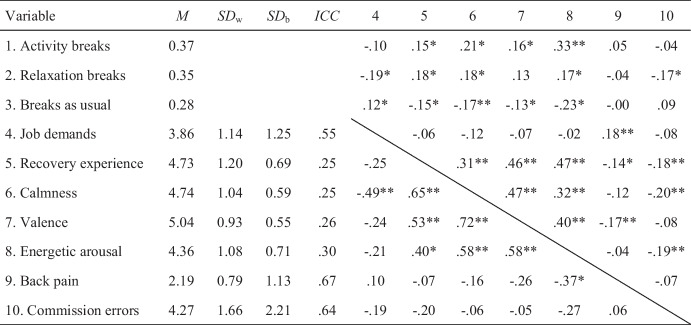
Descriptive statistics of the study variables

*N*_*2*_ = 34, *N*_*1*_ = 277

Above the diagonal, the within-person correlations are reported, and below the diagonal, the between-person correlations based on aggregated data are shown. *SD*_w_ = within-subject standard deviation. *SD*_b_ = between-subject standard deviation. *ICC* = intraclass correlation. For activity and relaxation breaks, the correlations refer to the reference category of breaks as usual

*p < .05; **p < .01

Calmness, valence and energetic arousal showed significant positive intercorrelations. Furthermore, calmness and energetic arousal were negatively correlated with the percentage of commission errors, while valence showed a significant negative correlation with back pain. To consider the correlations between the dependent variables, multilevel path models were estimated. Relaxation breaks, activity breaks and job demands were defined as within-subject variables with job demands being centered at the person-mean (Nezlek, 2011). Although the intercepts were allowed to vary randomly between the participants, the slopes were fixed to avoid unnecessary complications (Preacher et al., 2010).

Two models were estimated. The first model, the mediation model, presents the total effects of activity and relaxation breaks on calmness, valence, energetic arousal, back pain, and attention and the indirect associations mediated by the variable break recovery experience. We applied a Bayesian approach to account for the skewed sampling distribution of indirect effects (Preacher et al., 2010), and provide 95% credibility intervals (*CRI*) based on the posterior probability distribution. The model fit was checked by the posterior predictive *p*-value of the models and the confidence interval relating to the difference between the observed and generated data (Zyphur & Oswald, 2015). The total and indirect effects were requested by setting model constraints. Model constraints were also used to analyze the equality of the total effects for activity and relaxation breaks on the dependent variables.

The moderation model includes the main effects of activity and relaxation breaks and job demands and the interactions Activity Breaks x Job Demands and Relaxation Breaks x Job Demands. In the case of a significant interaction term, the simple slope of job demands on days with activity or relaxation breaks was estimated by changing the reference category. The originally specified model delivered the simple slope of job demands on days with breaks spent as usual, whereas the second model type delivered the simple slopes of job demands on days with activity or relaxation breaks.

## Results

### Total Associations of Activity Breaks and Relaxation Breaks with Mood, Back Pain and Attention and Indirect Associations Mediated by the Break Recovery Experience

Indicating good model fit (Zyphur & Oswald, 2015), the posterior predictive *p*-value of the model was greater than 0.01 (*PPP* = 0.26), and the confidence interval relating to the difference between the observed and generated data included zero (*CRI* 95% [–15.91, 55.06]. In line with hypotheses H1a and H2a, activity breaks and relaxation breaks are both significantly related with greater calmness after the break (activity breaks: estimate = 0.43, *CRI* 95% [0.11, 0.74]; relaxation breaks: 0.43, *CRI* 95% [0.10, 0.73]). These results suggest that the office workers experienced greater calmness on days with guided activity and relaxation breaks than on days on which they spent their breaks as usual. However, the data do not support hypotheses H1b and H2b, as in the context of the path model, there were no significant total associations of both break interventions with the mood dimension valence (activity breaks: estimate = 0.30, *CRI* 95% [-0.02, 0.60], relaxation breaks: estimate = 0.24, *CRI* 95% [-0.09, 0.52]). Supporting hypothesis H1c, we found a significant total association of activity breaks with energetic arousal (estimate = 0.74, *CRI* 95% [0.43, 1.03]), which was absent for relaxation breaks (estimate = 0.36, *CRI* 95% [-0.01, 0.69]; hypothesis H2c). However, on days on which the office workers engaged in guided relaxation breaks, they made fewer commission errors on the attention test than on days on which they spent their breaks as usual (estimate = -0.59, *CRI* 95% [-1.08, -0.11]). Activity breaks did not show such an association (estimate = -0.16, *CRI* 95% [-0.58, 0.28]). These results are in line with hypothesis H2d, but they do not support hypothesis H1d. Contrary to hypotheses H1e and H2e, both break interventions were unrelated to back pain (activity breaks: estimate = 0.09, *CRI* 95% [-0.17, 0.32]; relaxation breaks: -0.05, *CRI* 95% [-0.31, 0.20]).

By setting model constraints, we compared the size of the estimates for activity breaks and relaxation breaks. The results of these comparisons indicate that activity breaks show a stronger association with energetic arousal than relaxation breaks show (estimate = 0.39, *CRI* 95% [0.06, 0.68]). For the other dependent variables, there were no significant differences between the total associations of both break types (calmness: estimate = -0.02, *CRI* 95% [-0.31, 0.28]; valence: estimate = 0.07, *CRI* 95% [-0.18, 0.34]; attention: estimate = 0.43, *CRI* 95% [-0.02, 0.91]; back pain: estimate = 0.13, *CRI* 95% [-0.10, 0.37]).

Regarding the indirect associations of activity and relaxation breaks, we found strong support for the assumption that the break recovery experience constitutes an important mediator. First, activity breaks (estimate = 0.41, *CRI* 95% [0.03, 0.78]) and relaxation breaks (estimate = 0.46, *CRI* 95% [0.08, 0.85]) were both significantly associated with a more positive break recovery experience. Both coefficients did not differ from each other (estimate = -0.05, *CRI* 95% [-0.40, 0.26]). A positive recovery experience, in turn, related to greater calmness (estimate = 0.25, *CRI* 95% [0.14, 0.35]), valence (estimate = 0.34, *CRI* 95% [0.26, 0.43]), and energetic arousal (estimate = 0.40, *CRI* 95% [0.29, 0.50]) and lower values for back pain (estimate = -0.09, *CRI* 95% [-0.18, -0.01]) and the percentage of commission errors (estimate = -0.23, *CRI* 95% [-0.42, -0.07]). All indirect associations of both break interventions with the outcome variables of calmness, valence, energetic arousal, back pain, and the percentage of commission errors mediated by the recovery experience were significant (see Table 3). Thus, the data support hypotheses H3a-H3e and H4a-H4e.

**Table 3:** Unstandardized within-person path coefficients of the mediation model

	Activity breaks Estimate (*CRI*)	Relaxation breaks Estimate (*CRI*)
Indirect associations via break recovery experience		
Calmness	**0.09 [0.01, 0.22]**	**0.11 [0.02, 0.25]**
Valence	**0.14 [0.01, 0.27]**	**0.15 [0.03, 0.31]**
Energetic arousal	**0.16 [0.01, 0.32]**	**0.18 [0.03, 0.35]**
Back pain	**-0.03 [-0.10, -0.00]**	**-0.04 [-0.11, -0.00]**
Commission errors %	**-0.08 [-0.23, -0.02]**	**-0.10 [-0.25, -0.01]**
Total associations		
Calmness	**0.43 [0.11, 0.74]**	**0.43 [0.10, 0.73]**
Valence	0.30 [-0.02, 0.60]	0.24 [-0.09, 0.52]
Energetic arousal	**0.74 [0.43, 1.03]**	0.36 [-0.01, 0.69]
Back pain	0.09 [-0.17, 0.32]	-0.05 [-0.31, 0.20]
Commission errors %	-0.16 [-0.58, 0.28]	**-0.59 [-1.08, -0.11]**

Credibility intervals that do not contain 0 are in bold

The mediation model explained 3% of the variance of the mediator variable recovery break experience. The proportions of the explained variances of the dependent variables were between 5% and 27% (calmness: 12%, valence: 21%, energetic arousal: 27%, attention: 5%, back pain: 5%).

### Activity Breaks and Relaxation Breaks as Demand Buffers

The moderation model shows a good model fit (*PPP* = 0.22, *CRI* 95% [–12.45, 37.19]).

**Fig. 2:**
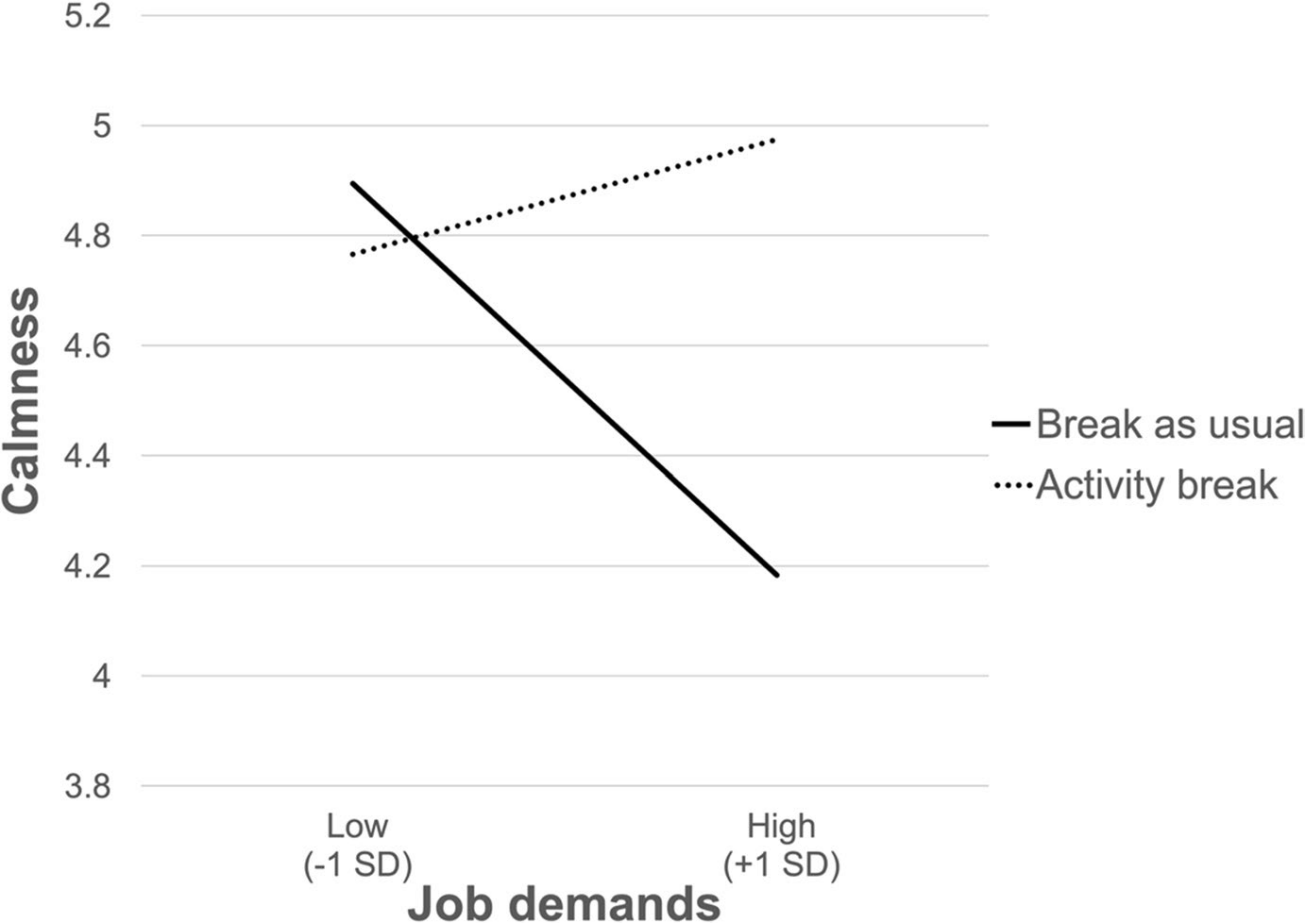
Moderating role of live-streaming activity breaks on the relationship between job demands and calmness

On control days, job demands were significantly associated only with calmness (estimate = -0.31, *CRI* 95% [–0.61, -0.05]) and back pain (estimate = 0.22, *CRI* 95% [0.01, 0.40]), and the relationships of job demands with the other dependent variables were absent (valence: estimate = -0.18, *CRI* 95% [–0.43, 0.06], energetic arousal: estimate = -0.10, *CRI* 95% [–0.36, 0.17]; attention: estimate = 0.40, *CRI* 95% [–0.88, 0.06]). Although activity breaks did not moderate the effects of job demands on energetic arousal (estimate = 0.21, *CRI* 95% [-0.11, 0.52]), attention (estimate = 0.24, *CRI* 95% [-0.31, 0.84]) or back pain (estimate = -0.11, *CRI* 95% [-0.35, 0.13]), activity breaks showed significant interactions with job demands on calmness (estimate = 0.41, *CRI* 95% [0.10, 0.76]) and valence (estimate = 0.31, *CRI* 95% [0.02, 0.58]). A comparison of the simple slopes showed that job demands were significantly associated with calmness on days on which breaks were spent as usual (estimate = -0.31, *CRI* 95% [–0.61, -0.05]), but they were unrelated to calmness on days with live-streaming activity breaks (estimate = 0.09, *CRI* 95% [–0.11, 0.29]). For the dependent variable of valence, the coefficient of job demands changed from estimate = -0.18 (*CRI* 95% [–0.43, 0.06]) under the condition of breaks as usual estimate = 0.12 (*CRI* 95% [–0.05, 0.29]) when the participants engaged in activity breaks. These results indicate that activity breaks may function as a buffer against the negative associations of job demands. The interaction of Activity Breaks x Job Demands is illustrated as an example for calmness in Fig. 2.

For relaxation breaks, however, no significant interactions with job demands were found (calmness: estimate = 0.16, *CRI* 95% [-0.18, 0.53]; valence: estimate = 0.03, *CRI* 95% [-0.26, 0.32]; energetic arousal: estimate = 0.03, *CRI* 95% [-0.35, 0.36]; back pain: estimate = -0.14, *CRI* 95% [-0.40, 0.12]; attention: estimate = 0.45, *CRI* 95% [-0.20, 1.10]). The moderation model explained 7% of the variance of the dependent variable of calmness, 5% of the variance of valence, 9% of the variance of energetic arousal, and 5% of the situational attention performance and the back pain ratings.

### Participant Feedback

In the feedback survey, the office workers were asked about their experiences of both break intervention types. Unfortunately, data from only 22 participants are available, as the remaining participants did not complete the feedback survey. On seven-point Likert scales from *strongly disagree* (1) to *strongly agree* (7), the participants stated whether they had enjoyed the activity breaks, whether they could imagine continuing to take part in activity breaks, and whether they would like to see short activity breaks offered on a permanent, voluntary basis. The same questions were asked for relaxation breaks. For both break types, the feedback from the participants was very positive. Activity breaks were rated *M* = 6.32 (*SD* = 1.36) for enjoyment and future participation and *M* = 6.27 (*SD* = 1.45) for a permanent offer of activity breaks. Relaxation breaks were also highly enjoyed (*M* = 6.00, *SD* = 1.80) and would be well received in the future and highly appreciated if implemented permanently (*M* = 5.95, *SD* = 1.73/1.79). When comparing the individual feedback for relaxation and activity breaks, a majority of the participants gave the same ratings (enjoyment: 59%, future participation: 64%, permanent offer: 73%). Of the remaining participants, six evaluated activity breaks more positively than relaxation breaks regarding enjoyment and future participation, while the reverse was true for three participants. Regarding a permanent offer, four participants preferred activity breaks over relaxation breaks, whereas two participants preferred relaxation breaks.

Additionally, the participants were asked whether they had liked the virtually guided format of the relaxation and activity breaks. With a mean of *M* = 6.23 (*SD* = 1.77), the participants strongly agreed with this question. Furthermore, all participants but one indicated that they preferred the virtually guided format over the person-guided activity and relaxation breaks.

## Discussion

### Summary and Discussion of the Study Results

Applying a within-subject design, the central aim of this study was to investigate the daily associations of live-streaming activity and relaxation breaks with affective, somatic and cognitive outcomes in the real working lives of office workers. We hypothesized that on days on which office workers engaged in live-streaming activity or relaxation breaks, calmness, valence, energetic arousal and attention would be higher and back pain would be lower after breaks than on days on which breaks were spent as usual. In line with the hypotheses, the virtually guided activity and relaxation breaks were positively associated with calmness compared to breaks spent as usual. Thus, both break types are helpful to start the second half of the working day in a relaxed mood. Associations of the break interventions with the valence dimension of mood, however, were only present in the form of a significant positive bivariate correlation between activity breaks and valence (relaxation breaks narrowly missed the significance level). In the path model that combines all variables, no significant association was found. The valence dimension showed significant positive correlations with calmness and energetic arousal, which were controlled in the path model. This indicates that the positive bivariate correlation between activity breaks and valence is based on the content-related overlap between valence and the other two mood dimensions.

In line with the hypothesis, activity breaks showed a positive association with energetic arousal. In the path model, the total effect of relaxation breaks on energetic arousal was just not significant, there was only a significant bivariate correlation between relaxation breaks and energetic arousal. The finding that activity breaks outperformed relaxation breaks regarding the mood subdimension of energetical activation corresponds to the results from the experimental study by Hoover et al. (2022), in which physical activity breaks were related to higher energy levels than relaxation breaks. Thus, physical activity may be particularly beneficial for replenishing energy resources.

However, regarding objectively measured attention, the office workers performed better on days in which they participated in a relaxation break than on days with regular break activities, but for activity breaks, such an association was absent. Thus, the hypothesis of a positive relationship with objective attention was supported for relaxation breaks, but not for activity breaks. The virtually guided relaxation breaks included mindfulness exercises, and empirical evidence indicates that such exercise improve selective and executive attention (Chiesa et al., 2011, for a review). Improvements in different attentional processes have been demonstrated for even brief mindfulness exercises (Zeidan et al., 2010). Assessing the capacity to inhibit automatic responses, the attention task used in this study may have been particularly sensitive to attentional improvements because of the mindfulness relaxation exercises (Heeren et al., 2009; Isbel et al., 2020), which we propose as an explanation for the superior effects of relaxation breaks over activity breaks on attention. In an experiment, Wollseiffen et al. (2016) showed a positive effect of short bouts of exercise on specific aspects of neurocognitive performance. While decision-making improved after a 3-min boxing break relative to a usual break or a break in a massage chair, attention and memory were not influenced. Thus, we assume that activity breaks may indeed benefit cognitive performance, but the effect may be limited to specific aspects of cognitive functioning. Prior empirical studies taking a within-subject intervention approach found that on days where participants engaged in increased walking activity during breaks (Sianoja et al., 2018) or when participants used an active workstation (Giumetti et al., 2021), employees reported better attention than when they spent their breaks as usual or engaged in usual desk work.

Contrary to the hypotheses, associations between activity and relaxation breaks and back pain were absent (as with the other symptoms whose results were not presented in detail). A plausible explanation is that the content of the activity and relaxation breaks varied between different breaks. While the exercises focused on the back on some activity-break days, the exercises addressed the mobilization of the whole body on other days. We assume that break exercises that repeatedly target specific symptoms could produce more conclusive results. Moreover, the items that assess somatic symptoms showed very low means, which indicates that compared to the states of low calmness, valence, and energetic arousal, the considered somatic symptoms were less relevant among the present sample of office workers. Furthermore, in contrast with the other dependent variables, somatic symptoms showed the largest proportion of between-subject variance. The intraclass correlation of 0.67 of the back pain item suggests that back pain is more chronic than mood states and may thus be more difficult to influence by a mild, low-threshold, and short-term break intervention. Giumetti et al. (2021) also failed to find differences in physical health symptoms between days on which employees worked at active workstations and days when they engaged in usual desk work, and they suggested that the effects of increased physical workplace activity may become apparent only with prolonged use.

We suggested that the break recovery experience plays a central mediating role in the associations of activity and relaxation breaks on the one hand and mood, attention, and back pain on the other hand. As expected, both break intervention types were associated with a more positive break recovery experience, which resulted as the central mediator of the associations of the activity and relaxation breaks and the dependent variables. The mediator break recovery experience was significantly related to calmness, valence, energetic arousal, back pain, and objectively measured attention, which emphasizes its crucial role for functioning at work. The finding that there were more positive indirect associations of both break interventions via break recovery experience than total associations indicates that the break recovery experience is indeed the most proximal outcome of activity and relaxation breaks^1^.

Interestingly, there was evidence that the live-streaming activity breaks functioned as a buffer against the negative associations between situationally elevated quantitative job demands and calmness/valence. The findings from this study are in line with those of Sawhney et al. (2018), who found that exercise as a recovery strategy moderated the relationship between occupational stress and mental health among firefighters, while for relaxation, such a buffer effect was absent. However, Grover et al. (2017) showed that among nurses, mindfulness reduced the positive association between emotional demands and psychological distress. We propose that guided relaxation exercises may lack a buffer function because they may be more difficult to effectively implement under high-stress conditions than physical activities, particularly for employees with little practice. Thus, while reaching a state of mindfulness should indeed function as a buffer when facing high demands, it may be more difficult to reach this state under stressful circumstances. Indeed, there is evidence from between-subject research that worry and rumination predict psychological disengagement from mindfulness interventions, reflected in a lower motivation and intent to practice, lower commitment and lower beliefs in its effectiveness in helping to deal with stressful situations (Banerjee et al., 2018).

Overall, the offer of the live-streaming break activities received very positive feedback from the participants, who thoroughly enjoyed the exercises, would participate in future break activities and would greatly appreciate a permanent offer. The positive evaluation of the participants is in line with the results by Bramante et al. (2018), who assessed supervisor and employee perspectives on incorporating 10-minute physical activity breaks into the workplace and found strong support for their desirability and feasibility.

### Practical Recommendations

The positive relationships of live-streaming activity and relaxation breaks with recovery, calmness, energetic arousal (for activity breaks only) and attention performance (for relaxation breaks only) and the very positive feedback from the participants lead to the conclusion that employers should explore ways to offer such guided break activities. While the participants highly appreciated the virtual format of the break exercises, the live sessions with fixed times were perceived as not flexible enough in a few cases. However, we believe that virtual live sessions at fixed times have some advantages over videos alone, as virtual live sessions include more direct social contact and allow flexible responses to spontaneous requests of participants. Furthermore, we assume that virtual live sessions may be more motivating in the context of regular participation in the long run. However, virtual live sessions clearly lack flexibility. The provision of the temporary recordings of daily break exercises could represent an ideal intermediate offer between virtual live sessions and single video recordings.

On the other hand, it is clear that virtually guided break activities are much more resource intensive than single videos. For small organizations and in occupational contexts where virtually guided break activities at fixed times are not readily implementable, a selection of videos of guided break exercises may represent an interesting alternative.

However, a further disadvantage of the format of virtually guided break activities is the limited break-related autonomy. Ideally, an offer of guided breaks would include some possibilities for participants to choose the timing and content according to their own preferences to support autonomy and person-break fit (Venz et al., 2019). In light of the balanced preferences regarding activity and relaxation breaks, it would be optimal to offer both types of break exercises daily if possible.

Interestingly, this study showed that guided activity breaks functioned as a buffer against the negative associations of job demands with calmness. In a situation of high time pressure, employees tend to react by working faster and working longer (Baethge et al., 2019). However, these are maladaptive coping strategies (Baethge et al., 2019). This study indicates that leaders should encourage their employees to take the time for break activities, especially under situational conditions of elevated stress.

The results of this study suggest that organizations would benefit from offering guided break activities because the activating and attention-promoting function of such activities is positively associated with performance. Based on this argumentation, employers should consider motivating employees to participate in guided break exercises by offering them outside standard break time as an additional paid break (Bramante et al., 2018; Sianoja et al., 2018). Generally, employers should encourage their employees to take adequately long breaks.

### Limitations and Future Research Directions

As it is common in the context of ambulatory assessment (Fisher & To, 2012), all constructs were measured by very short scales to avoid overburdening the participants. This procedure can be justified by the facts that situational constructs are often simpler and more concrete than their between-person counterparts, and that some sources of measurement error, such as response sets, are constant when taking a within-subject perspective (Fisher & To, 2012). When available, we chose instruments that were proven in the context of ambulatory assessment studies. However, regarding the within-subject reliability estimates, not all of the scales reached reliability estimates above 0.70. Due to the shortness of the scales, ambulatory assessment instruments will often not fulfill traditional standards (Calamia, 2019). In the majority of the published ambulatory assessment studies, the within-subject reliability estimates are not reported, and clear standards or rules of thumb are not available anyway (Calamia, 2019; Nezlek, 2017) argues in favor of more relaxed standards for experience sampling studies, but it remains unclear the degree to which the standards should be adapted (Calamia, 2019). Thus, it is difficult to evaluate whether all reliability estimates in this study are sufficient. Calamia (2019) draws attention to another important aspect: in experience sampling studies, reliability usually means internal consistency, and reliability estimates that are too high may not desirable because they may indicate low validity in terms of narrow facets of the target construct. Thus, “a moderate level of within-person reliability is likely preferable to a high level” (Calamia, 2019, p. 286). Tomko et al. (2014) regarded their within-subject internal consistency of 0.56 that referred to a four-item scale as “moderate”. As we worked with two to three items per scale, we generally believe that the reliability estimates for all constructs in our study are acceptable. However, compared with the other two-item scales, the valence dimension showed a rather low internal consistency; thus, the findings for this dimension should be regarded with caution.

The category of somatic symptoms was represented by a single item that refers to back pain. Interestingly, Matthews et al. (2022) recently showed that many single items in organizational research exhibit “very good or extensive validity, evidencing moderate to high content validity, no usability concerns, moderate to high test-retest reliability, and extensive criterion validity” (p. 639). Although these authors provide a single-item compendium for organizational psychology, the item that measures back pain is not validated for the context of ambulatory assessment. However, the back pain item shows face and content validity and reasonable within-subject correlations with job demands, the break recovery experience, and valence, which supports its applicability (Fisher & To, 2012).

Above, we stated that we assume virtual live sessions have benefits over videos alone. However, empirical data comparing different formats are absent. When an employer wants to offer guided breaks, what is the best practice, e.g., regarding the format and timing, for doing so? From a practical perspective, research answering these questions would be helpful.

As a manipulation check, the daily survey asked participants whether they had participated in each condition as planned. However, the survey did not ask for the reasons why they may have been unsuccessful. Future studies should investigate barriers to participation in break offers because knowledge about such factors would benefit break implementation.

While the total number of break intervention studies is limited, many of the past studies considered samples of office workers. In this study, all participants were computer workers, and the break exercises were targeted to this job profile. Therefore, caution is advised regarding the generalizability of the study findings to other occupations. We assume that organizational offers supporting break recovery are useful for a wide range of occupations when these offers take the special features of the sample and the context into account. In particular, employees form occupations with high job demands and other conditions that interfere with break recovery, such as permanent operational readiness, may greatly benefit from recovery-promoting break offers. We think of nurses and police task forces as examples.

Due to the complexity of multilevel path models, we did not include random effects (Preacher et al., 2010) and thus did not examine differences in the recovery potential of activity and relaxation breaks between the participants. However, it would be interesting to investigate whether some persons benefit more from break interventions than others and which individual characteristics these persons show. Such knowledge would not only be relevant from a theoretical perspective but would also be informative for the design of break offers, which should include useful exercises for all employees.

## Conclusion

The results of this study indicate that the daily calmness, energetic arousal, and attention performance of office workers may benefit from live-streaming break activities. The break recovery experience played a central mediating role in these associations, indicating that a better break recovery experience represents the most proximal benefit of activity and relaxation breaks. These results combined with the positive feedback from the participants support the recommendation for organizations to provide a permanent offer of virtually guided break activities, which represent a low-threshold, practicable, and flexible prevention measure. Interestingly, and consistent with the view that break activities may be seen as resources, it appeared that on days with situationally elevated job demands, virtually guided activity breaks functioned as a buffer against the negative associations of job demands with calmness and valence. Thus, especially under situational conditions of elevated stress, leaders should encourage their employees to take the time for recreative break activities.

## Data Availability

The data will not be deposited.

## Footnotes

1 While the indirect association solely represents the mediating paths via the break recovery experience, the total association refers to the overall extent to which a dependent variable is associated with activity or relaxation breaks. The total association represents the sum of all direct and indirect associations. For example, we found no overall associations between both break activities and valence. However, the path model supported indirect associations of activity and relaxation breaks via the mediator break recovery experience. This means both break activities are indirectly related to higher valence because they are related to a better break recovery experience, which in turn predicts higher valence.
